# Carbon Storage Response to Land Use/Land Cover Changes and SSP‐RCP Scenarios Simulation: A Case Study in Yunnan Province, China

**DOI:** 10.1002/ece3.70780

**Published:** 2025-01-08

**Authors:** Jing Liu, Kun Yang, Shaohua Zhang, Wenxia Zeng, Xiaofang Yang, Yan Rao, Yan Ma, Changyou Bi

**Affiliations:** ^1^ Faculty of Geography Yunnan Normal University Kunming China; ^2^ GIS Technology Research Center of Resource and Environment in Western China, Ministry of Education Yunnan Normal University Kunming China; ^3^ Southwest United Graduate School Kunming China

**Keywords:** Carbon storage, InVEST, LULC, PLUS, SSP‐RCP

## Abstract

Changes in terrestrial ecosystem carbon storage (CS) affect the global carbon cycle, thereby influencing global climate change. Land use/land cover (LULC) shifts are key drivers of CS changes, making it crucial to predict their impact on CS for low‐carbon development. Most studies model future LULC by adjusting change proportions, leading to overly subjective simulations. We integrated the Integrated Valuation of Ecosystem Services and Trade‐offs (InVEST) model, the Patch‐generating Land Use Simulation (PLUS) model, and the Land Use Harmonization 2 (LUH2) dataset to simulate future LULC in Yunnan under different SSP‐RCP scenarios of climate and economic development. Within the new PLUS‐InVEST‐LUH2 framework, we systematically analyzed LULC alterations and their effects on CS from 1980 to 2040. Results demonstrated that: (1) Forestland had the highest CS, whereas built‐up land and water showed minimal levels. Western areas boast higher CS, while the east has lower. From 1980 to 2020, CS continuously decreased by 29.55 Tg. In the wake of population increase and economic advancement, the area of built‐up land expanded by 2.75 times. Built‐up land encroaches on other land categories and is a key cause of the reduction in CS. (2) From 2020 to 2040, mainly due to an increase in forestland, CS rose to 3934.65 Tg under the SSP1‐2.6 scenario, whereas under the SSP2‐4.5 scenario, primarily due to a reduction in forestland and grassland areas, CS declined to 3800.86 Tg. (3) Forestland is the primary contributor to CS, whereas the ongoing enlargement of built‐up land is causing a sustained decline in CS. Scenario simulations indicate that future LULC changes under different scenarios will have a significant impact on CS in Yunnan. Under a green sustainable development pathway, Yunnan can exhibit significant carbon sink potential. Overall, this research offers a scientific reference for optimizing land management and sustainable development in Yunnan, aiding China's “double carbon” goals.

## Graphical abstract

We integrated the InVEST model, PLUS model, and LUH2 dataset to simulate future LULC patterns under different SSP‐RCP scenarios. Within the framework, we took Yunnan Province as the study area and systematically analyzed the LULC alterations and their effects on CS from 1980 to 2040.

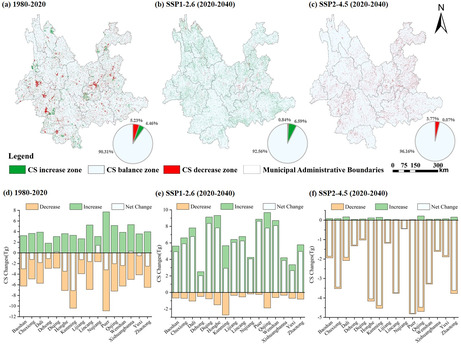

## 1 Introduction

Carbon storage (CS) is crucial to global carbon cycling and climate change (Houghton 2003; Tang et al. 2018). Increasing the CS can lower atmospheric CO_2_ levels, thus mitigating global climate change (Filonchyk et al. 2024; Yoro and Daramola 2020). According to the Intergovernmental Panel on Climate Change (IPCC) report, carbon emissions due to shifts in land use/land cover (LULC) are the second greatest atmospheric carbon source, surpassed only by fossil energy consumption (Houghton 1999; Lambin et al. 2001). However, LULC changes are the main and fundamental manifestation of the interplay between human actions and the ecosystem (Canadell and Raupach 2008). Such changes reshape the ecosystem, consequently impacting the carbon cycle and leading to variations in CS. (Li et al. 2021; Li et al. 2020; Li, Liu, and Feng 2022; Zeng et al. 2022). As a result, LULC changes are one of the most direct human‐driven factors affecting the carbon cycling (Piao et al. 2018; Yang et al. 2019). As the world's second‐largest economy, China is one of the largest carbon‐emitting countries globally (Hong et al. 2021; Houghton 1999). Existing studies indicate that the CS in most regions of China are declining (Liu et al. 2019; Wang et al. 2021). Therefore, exploring regional historical and future LULC changes and their impacts on CS (Cannell et al. 1999; He et al. 2016; Houghton 2001; Vizcaíno‐Bravo, Williams‐Linera, and Asbjornsen 2020) can provide a foundation for optimizing and adjusting LULC to enhance CS, encourage low‐emission growth, and facilitate the achievement of the “dual carbon” goals of China (Chen and Ma 2022; Li, Li, and Wang 2019).

Coupling CS estimation models with LULC prediction models is currently the primary method for exploring regional LULC changes effects on CS in terrestrial ecosystems (Yang et al. 2023). The Integrated Valuation of Ecosystem Services and Tradeoffs (InVEST) model uses a distributed algorithm that can quickly and accurately visualize the spatiotemporal distribution of CS (Wang, Mo, et al. 2024). It features benefits like uncomplicated operation, flexible parameters and high precision. It has been widely utilized to estimate CS by numerous scholars in various regions from different viewpoints and scales (Islam et al. 2022; Wang, Li, and Jin 2024; Zhu et al. 2022). The Patch‐generating Land Use Simulation (PLUS) model is frequently used for future LULC simulations because it can accurately simulate patch‐level shifts in multiple land classes for a wide range of applications (Gao et al. 2022; Li, Gong, and Guldmann 2022; Li, Liu, and Li 2022). Combining the InVEST model and the PLUS model to analyze historical and future alterations in regional LULC and CS under various scenarios is a valid method for estimating the effect of regional LULC changes on CS (Gong et al. 2023; Xiang et al. 2022).

LULC changes and their impact on CS are influenced by climate change and societal progress trends (Mottl et al. 2021). However, most existing studies subjectively adjust the proportions of LULC change to set scenarios that prioritize ecological conservation, cropland preservation, or economic development, and such simulations are overly subjective (Wei, Kasimu, et al. 2023; Zhang and Zhang 2023). These scenarios are based on historical LULC changes, natural and socio‐economic factors to predict future LULC and their effect on CS. These studies overlook the impact of LULC changes on CS under different future climate and socioeconomic development contexts. Shared Socioeconomic Pathways (SSPs) and Representative Concentration Pathways (RCPs) offer a spectrum of potential future development scenarios in the face of global climate change (Cook et al. 2020; Eyring et al. 2016). SSP‐RCP scenarios have been extensively utilized to forecast future LULC changes. Land Use Harmonization 2 (LUH2) dataset offers global LULC information under future SSP‐RCP scenarios (Hurtt et al. 2020; Liao et al. 2020). There have been studies using LUH2 data to predict future LULC under various SSP‐RCP scenarios at different regional scales, including China (Guo et al. 2024), the Yellow River Basin (Wang, Wu, et al. 2024), and Hainan Province (Wu et al. 2024). Therefore, we used the PLUS‐InVEST‐LUH2 framework to predict future LULC changes and their impacts on CS under SSP‐RCP scenarios. It provides theoretical references for achieving China's “dual carbon” goals.

As an ecological security barrier in southwest China, Yunnan Province has abundant forest resources, diverse species, and stable ecosystems. Its terrestrial ecosystems have a strong carbon sequestration capacity, making it an important carbon sink region in China (Cai et al. 2022; Peng et al. 2023; Zhang, Zhao, and Wu 2023). In studies on CS in Yunnan Province, most focus on estimating forest CS. Cheng et al. (2024) estimated forest carbon stocks based on a detailed land use classification of Yunnan Province. Zhou et al. (2016) and Tu et al. (2023) calculated forest carbon stocks using the inventory data of forest resources in Yunnan Province. Zhou et al. (2018) predicted the potential distribution of major forest vegetation types in Yunnan and estimated changes in carbon stocks and carbon sequestration potential with rising temperatures. But forest CS is only a part of the terrestrial ecosystem carbon stocks. Although the carbon sequestration capacity of non‐forest ecosystems, such as croplands and grasslands, is lower than that of forest ecosystems, they still play a key role in the global terrestrial carbon cycle (Liu et al. 2022; Scurlock and Hall 1998). Furthermore, non‐forest ecosystems cover a vast area and are widely distributed across Yunnan Province, making them an important carbon storage reservoir in the province's terrestrial ecosystems. Existing studies also focus heavily on ecosystem services (Sun et al. 2023; Zhang, Liao, and Zhai 2018) in Yunnan Province. In these studies, the CS is often only briefly analyzed as one indicator to assess ecosystem services, which is insufficient for systematically revealing the LULC changes and their impacts on CS. Based on the above points, we selected Yunnan Province as the study area. Under the PLUS‐InVEST‐LUH2 framework, we simulated the future LULC and CS in Yunnan Province under different SSP‐RCP scenarios and systematically analyzed LULC changes from 1980 to 2040 and their impacts on CS. Specific objectives include: (1) Analyzing the geographical traits of LULC and CS from 1980 to 2020 in Yunnan Province. (2) Simulate and predict the spatiotemporal evolution of LULC and CS under integrated scenarios of future climate change and economic development. (3) Investigating how LULC changes affect CS.

## 2 Materials and Methods

### 2.1 Study Area

**FIGURE 1:**
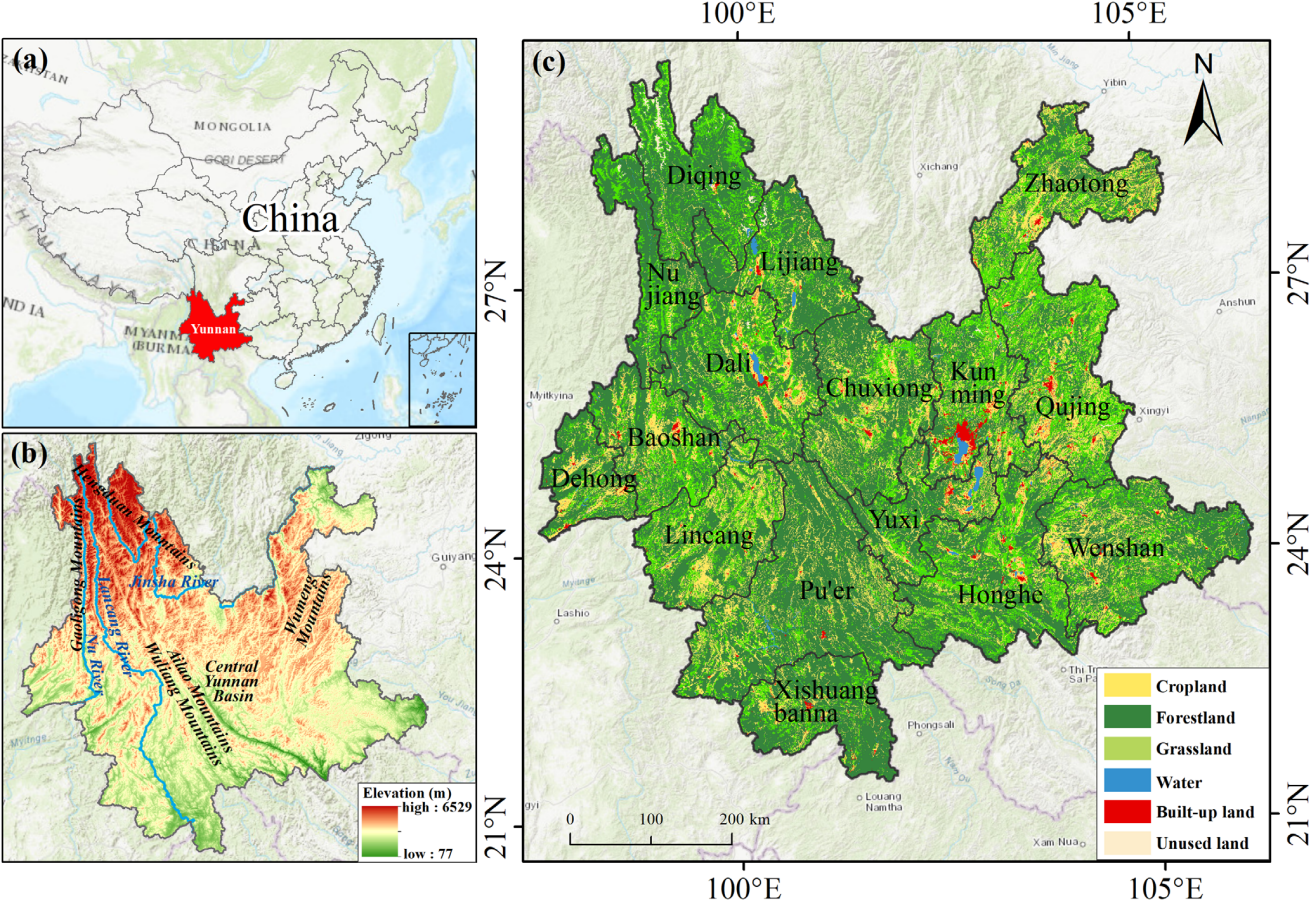
The geographical position and environment of Yunnan Province. (a) location of Yunnan Province within China. (b) Digital Elevation Model (DEM) of Yunnan Province. (c) land use map of Yunnan Province in 2020, along with the administrative divisions at the prefecture level.

Yunnan Province (97°31′‐106°11′ E, 21°8′‐29°15′ N) is a border province with complex and diverse natural conditions, located in the southwestern frontier of China (Figure 1). Yunnan features a mountainous plateau terrain, with mountains, hills, and plateaus making up 94% of its land. Its topography declines step by step from north to south. By the influence of the special topography and geomorphology of Yunnan Province has formed complex climate characteristics. Yunnan belongs to the subtropical and tropical monsoon climate, which features distinct dry and wet seasons and significant vertical climate characteristics (Yang et al. 2004). Yunnan Province has extensive forest areas, rich biodiversity, and supportive forestry policies, and it is an important region for future carbon sink enhancement in China (Qian et al. 2020). Yunnan Province's population grew from 31.73 to 47.22 million, and the provincial GDP rose from 8.4 billion yuan to 2.46 trillion yuan during the period from 1980 to 2020.

### 2.2 Data Sources

We used 30 m resolution historical LULC data and 0.25° resolution future LULC data based on SSP‐RCP scenarios. We selected 16 key drivers influencing LULC changes, including natural and socio‐economic factors. The carbon density data for calculating CS was obtained from *Global Aboveground and Belowground Biomass Carbon Density Maps for the Year 2010* of Oak Ridge (Spawn and Gibbs 2020) and the *Global Soil Organic Carbon Map* (GSOCmap) of the FAO. The distances to various roads, water, and governments were computed using the Euclidean distance method. Table 1 shows the sources of each dataset. All data were preprocessed in ArcGIS 10.8 with projection transformation, cropping, resampling, etc. They were standardized to the same projection coordinate system (WGS_1984_UTM_Zone_47N), and the raster data were resampled to 100 m resolution.

**TABLE 1:** Data sources.

Category	Data	Time	Source
Land use	CNLUCC datasets	1980–2020	Resource and Environmental Science Data Center of the Chinese Academy of Sciences (https://www.resdc.cn/)
	LUH2 data	2030, 2040	Land‐Use Harmonization (https://luh.umd.edu/)
Natural factors	Precipitation	2020	National Tibetan Plateau/Third Pole Environment Data Center (https://data.tpdc.ac.cn/)
	Temperature	2020	
	DEM		Geospatial Data Cloud site (https://www.gscloud.cn/)
	Slope		Extraction based on DEM data
	Soil type	1995	Resource and Environmental Science Data Center of the Chinese Academy of Sciences (https://www.resdc.cn/)
	Soil erosion	1995	
Socioeconomic factors	Nighttime light	2020	
	GDP	2019	
	Population density	2020	Oak Ridge (https://landscan.ornl.gov/)
Road, river, and city networks	Distance to highways		Open Street Map (https://www.openstreetmap.org/)
	Distance to first roads		
	Distance to secondary roads		
	Distance to third roads		
	Distance to railways		
	Distance to water		
	Distance to governments	2022	National Bureau of Statistics (https://www.stats.gov.cn/)
Carbon density	*C*_above_, *C*_below_	2010	Oak Ridge (https://doi.org/10.3334/ORNLDAAC/1763)
	*C*_soil_		Food and Agriculture Organization of the United Nations (https://www.fao.org/home/en/)

### 2.3 Methods

#### 2.3.1 Dynamic LULC

The land use transfer matrix can express the direction and quantity of reciprocal transfers between various LULC categories within the study region throughout the study timeframe. It facilitates the understanding of general LULC dynamics and the alterations in the structure of LULC (Wu, Luo, et al. 2022). Its basic expression (1) is as follows:

(1) $$S_{\mathrm{ij}}=\left[\begin{array}{ccc} S_{11} & \ldots & S_{1n}\\ \ldots & \ldots & \ldots \\ S_{n1} & \ldots & S_{\mathrm{nn}} \end{array}\right]$$

In this equation, $S_{\mathrm{ij}}$ represents the initial LULC type i converted to the ending type j; n embodies the quantity of LULC types.

#### 2.3.2 InVEST Model

The InVEST model is an open‐source ecosystem service assessment (Cao et al. 2019; Kupfer 2012; Yang et al. 2022). We can use its carbon storage and sequestration module to calculate CS by entering LULC raster and carbon density data (Wang, Li, and Li 2022). The calculation formulas (2) and (3) are presented below:

(2) $$C_{i}=C_{i,\text{above}}+C_{i,\text{below}}+C_{i,\text{soil}}+C_{i,\text{dead}}$$

(3) $$C_{\text{total}}=\sum_{i=1}^{n}C_{i}\times S_{i}$$

Where i represents the LULC type; $C_{i}$ is the carbon density of the LULC type; $C_{\text{above}}$, $C_{\text{below}}$, $C_{\text{soil}}$, $C_{\text{dead}}$ denote the above‐ground biocarbon density, below‐ground biocarbon density, soil organic carbon density, and dead organic carbon density (*t*/ha) of the LULC type, respectively; $C_{\text{total}}$ is the total CS (*t*); $S_{i}$ is the area of LULC type i (km^2^); n is the number of LULC types, which is 6 in this study.

The $C_{\text{above}}$, $C_{\text{below}}$ and $C_{\text{soil}}$ for LULC type were obtained from *Global Aboveground and Belowground Biomass Carbon Density Maps for the Year* 2010 and *GSOCmap*. Since $C_{\text{dead}}$ mainly consists of the carbon density of apoplastic material, etc., which is difficult to measure and obtain (Nie et al. 2020). So, we set it to 0. Final carbon densities for all land classifications in Yunnan Province are displayed in Table 2.

**TABLE 2:** Average carbon density of each LULC type in Yunnan (*t*/ha).

Land use type	*C*_above_	*C*_below_	*C*_soil_	*C*_dead_
Cropland	13.19	4.31	50.10	0.00
Forestland	48.14	15.49	57.97	0.00
Grassland	21.86	7.96	51.97	0.00
Water	0.00	0.00	0.00	0.00
Built‐up land	0.00	0.00	0.00	0.00
Unused land	0.74	1.91	61.47	0.00

#### 2.3.3 PLUS Model

The PLUS model integrates the land expansion analysis strategy (LEAS) module and the cellular automata‐based on multiple random patch seed (CARS) module (Liang et al. 2021). It can more accurately simulate the formation and development of land patches (Guo et al. 2022; Lin and Peng 2022). To verify the model's accuracy, we employed LULC data from 2000 and 2010 to simulate 2020. Subsequently, we used the “Confusion Matrix and FOM” accuracy validation module integrated into the model to compare the simulated 2020 LULC data with the actual 2020 LULC data, obtaining a Kappa coefficient of 0.92, an FOM accuracy of 0.60, and an overall accuracy of 0.95. Therefore, the simulation results are highly dependable, and the model is suitable for future LULC simulations.

#### 2.3.4 Future LULC Predictions Based on LUH2

The LUH2 dataset offers global historical and future LULC data between 2015 and 2100, covering eight scenarios that combine five SSPs and seven RCP targets (Liao et al. 2020). We used LUH2 data to establish scenarios and predict future LULC. Taking into account potential future development trends, we chose the SSP1‐2.6 and SSP2‐4.5 scenarios from the eight future scenarios. The SSP1‐2.6 scenario represents a future development pathway characterized by low greenhouse gas emissions, high economic growth, rapid technological advancement, low population growth, and strong policy influence (Guan et al. 2021). It highlights the preservation of ecosystems and the pursuit of environmentally sustainable growth. The SSP2‐4.5 scenario reflects moderate economic growth, technological advancement, population growth, and low policy influence, resulting in moderate greenhouse gas emissions (Wu, Ding, et al. 2022). The scenario emphasizes the continuation of current trends with a stable socio‐economic system.

Using high‐resolution initial LULC maps to simulate can enhance the accuracy in the PLUS model. Initially, we computed the future rate of LULC area transformation for all types based on LUH2 data and got the future area demands through correction (Table A). Then, we used the 2020 LULC data of Yunnan Province at a resolution of 100 m as the initial data and combined it with various drivers to simulate the future data.

## 3 Results

### 3.1 LULC Changes Characteristics and SSP‐RCP Scenarios Simulation in Yunnan Province

#### 3.1.1 LULC Changes Characteristics From 1980 to 2020

**FIGURE 2:**
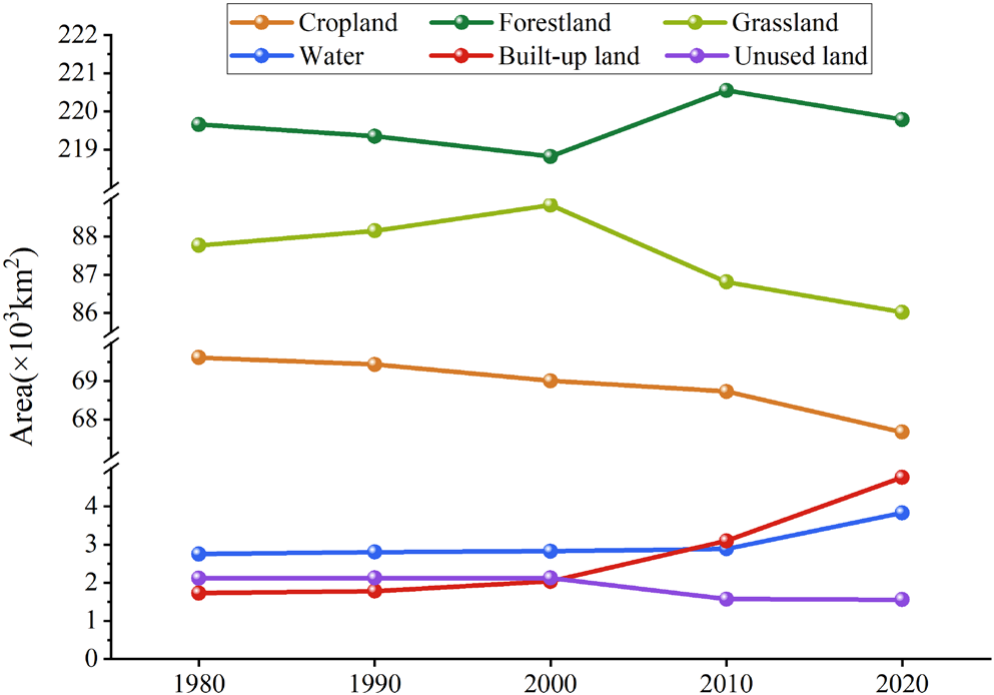
Changes in the area of various LULC types from 1980 to 2020.

Between 1980 and 2020, the LULC structure in Yunnan Province has been dominated by forestland, grassland, and cropland, which together accounted for over 97% of the total area (Figure 2). Forestland accounts for 57% of the area in Yunnan. From 2000 to 2010, forestland increased by approximately 1730 km^2^. In other periods, forestland decreased, but the amount of decrease was relatively small. As a result, the area of forestland in 2020 has overall risen by 130 km^2^ compared to 1980. Grassland, respectively accounted for 22.88%, 22.98%, 23.15%, 22.63%, and 22.42% of the total area in 1980, 1990, 2000, 2010, and 2020, demonstrating an initial boost followed by a fall, the overall reduction amounts to 1750 km^2^ due to the larger decrease in the latter 20 years. From 1980 to 2020, cropland continuously decreased, with a total reduction of approximately 1950 km^2^. This decline was especially pronounced between 2010 and 2020, during which about 1070 km^2^ was lost. Between 1980 and 2020, the share of built‐up land rose from 0.45% to 1.24%, with a total increase of 3030 km^2^. Although its overall proportion is relatively small, it experienced the most significant change among all land categories. Particularly, built‐up land rose by 133.33% from 2000 to 2020. From 1980 to 2020, water's area continuously increased, with a significant increase of about 940 km^2^ between 2010 and 2020. The total change in unused land is not significant, showing only a slight decline. However, between 2000 and 2010, the change in unused land was relatively large. The variations in LULC areas for different types from 1980 to 2020 (absolute values) are as follows: built‐up land>cropland > grassland > water > unused land > forestland.

**FIGURE 3:**
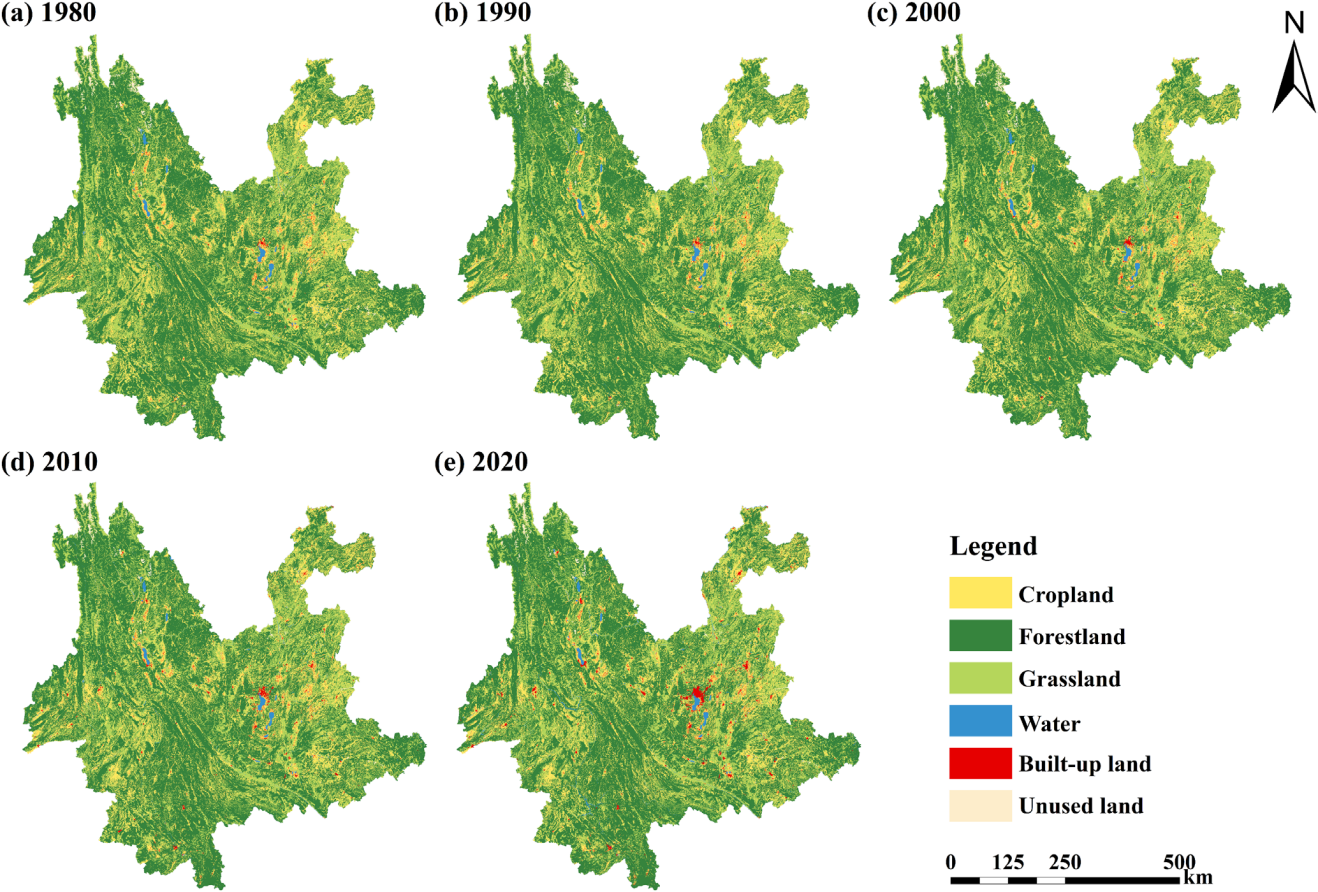
Yunnan Province's LULC type distribution from 1980 to 2020. (a–e) represent the LULC type distribution in Yunnan Province for the years 1980, 1990, 2000, 2010, and 2020, respectively.

The spatial distribution of various LULC types did not undergo significant changes from 1980 to 2020 in Yunnan Province (Figure 3). Forestland is more extensively dispersed in the northern mountainous and southern tropical areas. Grassland and forestland are interspersed, with grassland being more extensively distributed in the eastern areas. Cropland is widely distributed in the basins of the eastern and southeastern and valleys regions. Built‐up land is typically established in the relatively level regions. Water mainly consists of Yunnan's nine major plateau lakes, the Jinsha River, Lancang River, Nu River, and areas used for water conservancy facilities. Unused land is widely dispersed throughout high‐altitude regions of Diqing.

**FIGURE 4:**
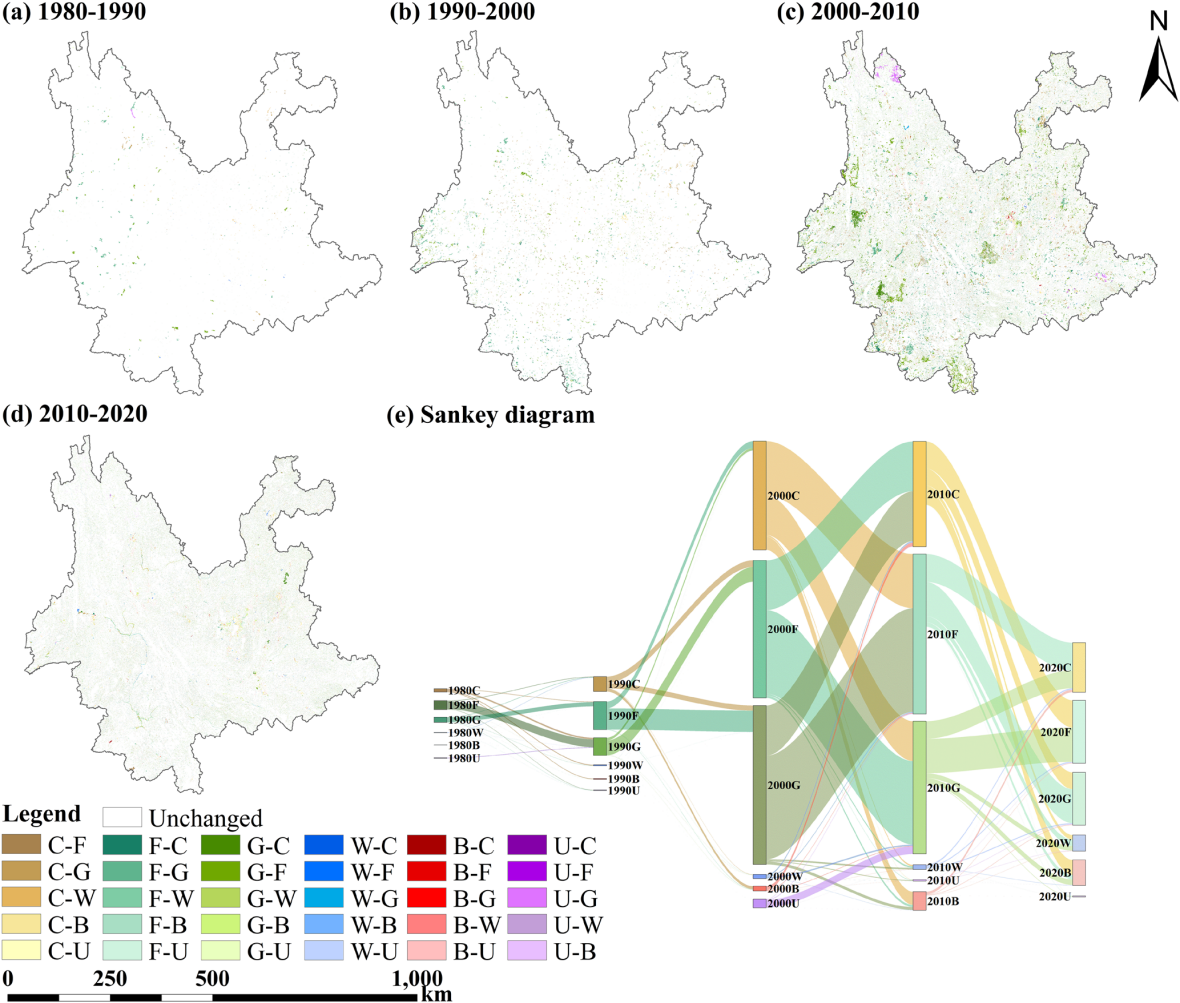
LULC types conversion in Yunnan Province from 1980 to 2020. (a–d) are the spatial distributions of LULC type conversions, and (e) is the Sankey diagram of LULC type conversions.

Due to the absence of large contiguous areas of LULC changes, LULC changes are spatially dispersed, making the LULC changes in Yunnan Province appear relatively stable in terms of geographic distribution. However, in terms of area, LULC changes do exist, and the mutual conversions between various LULC categories are significant (Figure 4). The flow of changes between various LULC categories in different eras was represented through a Sankey diagram (Figure 4e). From 1980 to 2020, the largest total area of transitions occurred during the period from 2000 to 2010. Because forestland, grassland, and cropland cover vast areas in Yunnan Province, the area of mutual conversion between these three land types is also the largest. From 1980 to 2020, the largest area of mutual conversion occurred between forestland and grassland. In the first 20 years, there was more conversion from forestland to grassland, while in the latter 20 years, there was more conversion from grassland to forestland. Especially during the period from 2000 to 2010, there were 8012.16 km^2^ of grassland converted to forestland, with significant areas in Nujiang and the southern part of Xishuangbanna. From 1980 to 2020, the inflow of cropland has consistently been less than the outflow. In addition to the mutual conversion with forestland and grassland, a sizable portion of cropland was also turned into built‐up land. Between 2000 and 2010, significant areas of grassland in central Baoshan and southern Lincang were converted into cropland. The shift from cropland to built‐up areas is showing an upward trend. Between 2000 and 2020, the total area of cropland transformed into built‐up land reached 2169.58 km^2^. The phenomenon of built‐up land encroaching on cropland around existing construction areas has become more pronounced. Water primarily increased around certain sections of rivers like the Lancang River, with conversions from cropland, forestland, and grassland. Unused land is mainly converted into grassland, with significant areas of bare rocky terrain in northern Diqing transformed into grassland.

#### 3.1.2 SSP‐RCP Scenarios LULC Projections for 2030 and 2040

We used the PLUS model and LUH2 dataset to simulate the LULC in 2030 and 2040 under SSP‐RCP scenarios in Yunnan Province. The results show that there are significant differences in the future LULC changes in Yunnan Province under the two different scenarios. In the SSP1‐2.6 scenario, changes in grassland, cropland, unused land, and forestland are more significant compared to the SSP2‐4.5 scenario (Table 3). Specifically, grassland decreased by 0.29% (24.63 × 10^3^ km^2^), built‐up land increased by 0.28% (1.33 × 10^3^ km^2^), unused land initially declined and then rose, causing an overall rise of 0.13% (0.20 × 10^3^ km^2^), and forestland increased by 0.11% (25.02 × 10^3^ km^2^). Contrary to the SSP1‐2.6 scenario, the most major variation is in cropland in the SSP2‐4.5 scenario. Cropland rose by 0.20% (13.26 × 10^3^ km^2^), forestland decreased by 0.02% (4.10 × 10^3^ km^2^), and unused land experienced a slight reduction. Comparing the two scenarios, the future LULC changes in the SSP1‐2.6 scenario are more dramatic, likely due to the implementation of stringent ecological protection policies and rapid economic development. In contrast, the SSP2‐4.5 scenario represents a more moderate development path, with weaker policy constraints and slower economic growth, resulting in relatively modest LULC changes in the future.

**TABLE 3:** Change in LULC type in 2030 and 2040 under 2 different scenarios relative to that in 2020.

	Land use type	SSP1‐2.6		SSP2‐4.5	
		2030	2040	2030	2040
Area change (×10^3^ km^2^)	Cropland	−3.31	−1.92	8.57	13.26
	Forestland	5.94	25.02	−2.84	−4.10
	Grassland	−3.41	−24.63	−6.29	−10.07
	Built‐up land	0.80	1.33	0.56	0.93
	Unused land	−0.01	0.20	0.00	−0.02
Proportion change (%)	Cropland	−0.05	−0.03	0.13	0.20
	Forestland	0.03	0.11	−0.01	−0.02
	Grassland	−0.04	−0.29	−0.07	−0.12
	Built‐up land	0.17	0.28	0.12	0.19
	Unused land	−0.01	0.13	0.00	−0.01

**FIGURE 5:**
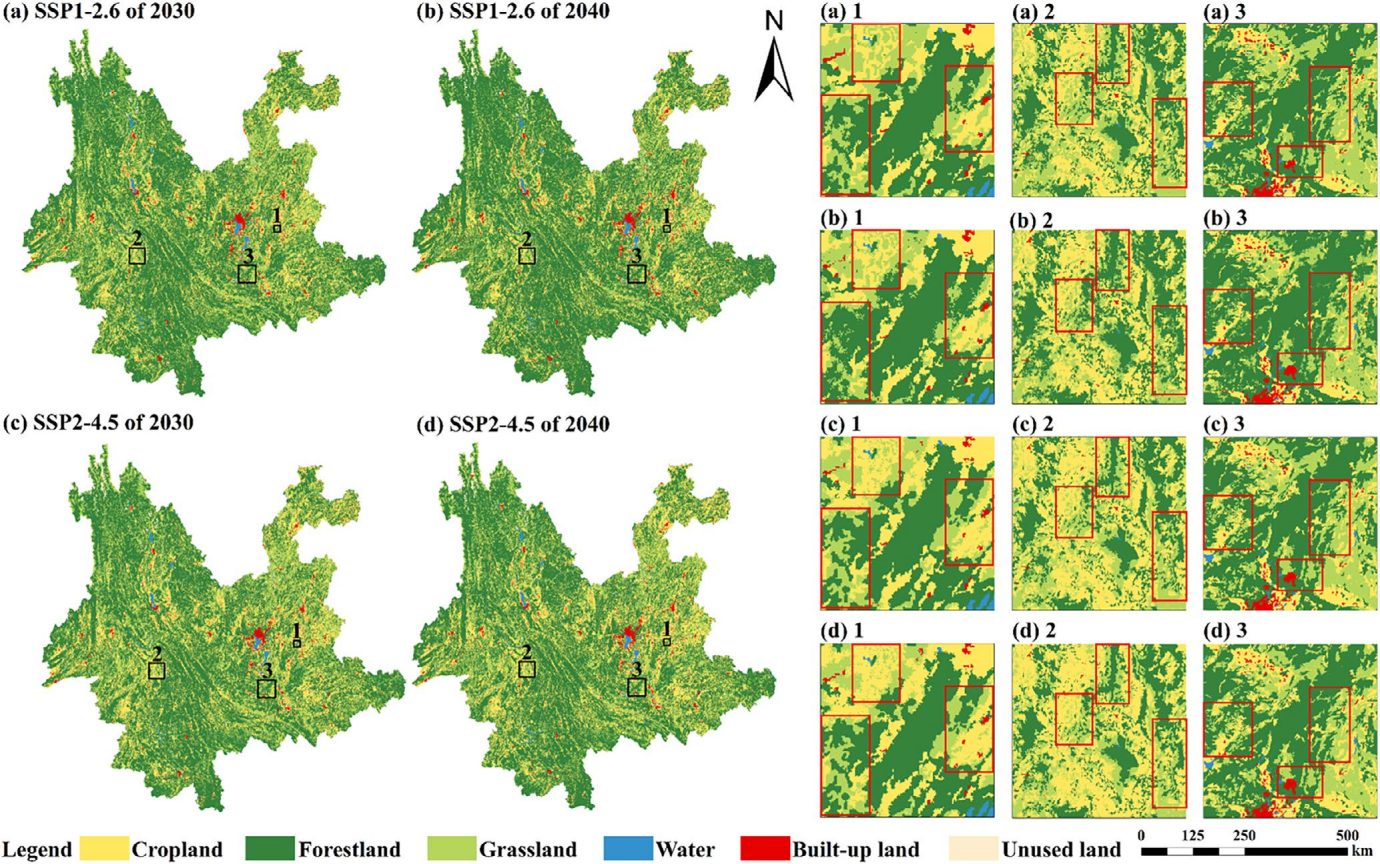
Spatial distribution of LULC types in Yunnan Province from 2030 to 2040. (a–d) represent the LULC in Yunnan Province for 2030 and 2040 under the SSP1‐2.6 and SSP2‐4.5 scenarios, respectively. (a) 1 indicates the zoomed‐in view of the rectangular box 1 in (a). (b) 1 indicates the zoomed‐in view of the rectangular box 1 in (b). Similarly, (a) 1‐ (d) 3 refer to zoomed‐in views in their respective figures.

The LULC geographical distribution in the two forecasted scenarios is generally consistent with the historical patterns (Figure 5). Under the two scenarios, the built‐up land continues to expand into neighboring areas, especially in the central Yunnan urban region. The forestland area in Yunnan Province continues to increase significantly, while grassland continues to decrease, with most of the grassland being converted into forestland in the SSP1‐2.6 scenario. The forestland is expanding around the existing forestland areas. In the SSP2‐4.5 scenario, apart from a rise in cropland area, the land type distribution in 2030 and 2040 resembles that of 2020, without notable changes.

### 3.2 CS Dynamics in Yunnan Province From 1980 to 2040

**FIGURE 6:**
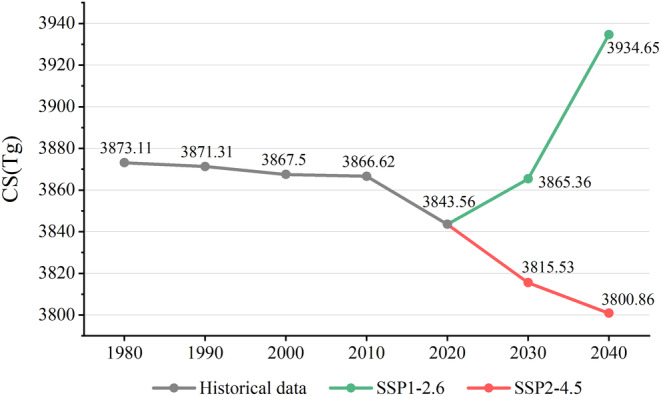
Changes in CS in Yunnan Province from 1980 to 2040.

We employed the InVEST model to estimate CS in Yunnan between 1980 to 2040, relying on LULC data and carbon density data associated with each class (Figure 6). The results show that CS decreased continuously from 1980 to 2020 in Yunnan, with an overall reduction of 29.55 Tg(10^6^tone). The decrease was less from 1980 to 2010, but more pronounced from 2010 to 2020, amounting to approximately 23.05 Tg, which accounts for 78% of the total decrease over the four decades.

From 2020 to 2040, the CS in Yunnan Province shows a continuous upward trend under the SSP1‐2.6 scenario, while they exhibit an ongoing downward movement under the SSP2‐4.5 scenario. The CS shows significant growth in the SSP1‐2.6 scenario, with gains of 22 Tg and 91 Tg in 2030 and 2040, respectively. Under this scenario, the growth of high‐carbon‐density forestland contributes substantially to the increase in CS. In the SSP2‐4.5 scenario, the CS decreases by 28 Tg and 43 Tg in 2030 and 2040, respectively. In this scenario, not only does the increase in low‐carbon‐density built‐up land occur, but there is also a reduction in high‐carbon‐density forestland and grassland, which together lead to the decline in CS.

**FIGURE 7:**
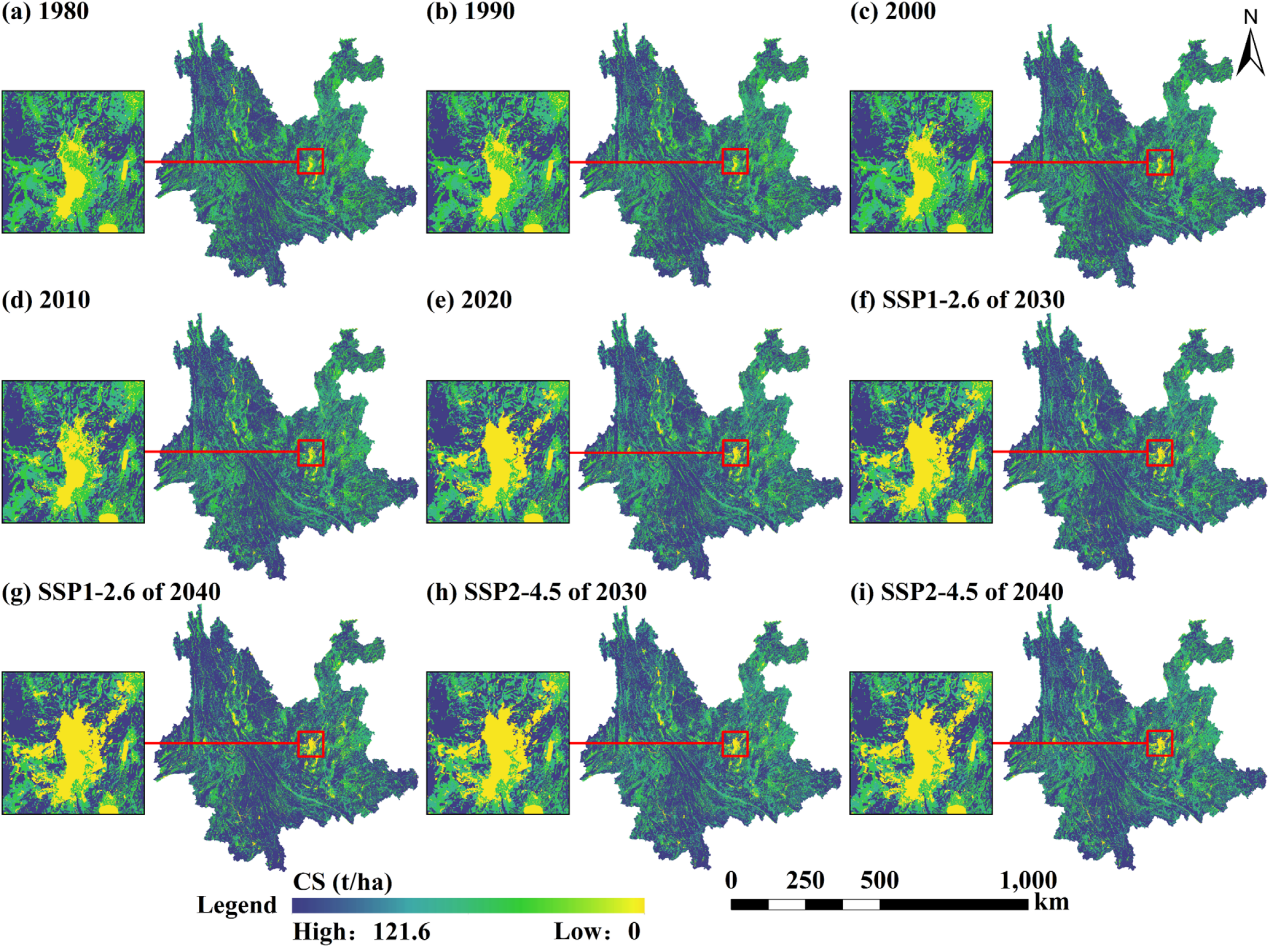
Spatial distribution of CS in Yunnan Province from 1980 to 2040. (a–e) show the CS distribution in Yunnan Province for 1980, 1990, 2000, 2010, and 2020, respectively. (f–i) show the CS distribution for 2030 and 2040 under the SSP1‐2.6 and SSP2‐4.5 scenarios, respectively. The rectangular boxes on the left of each figure represent zoomed‐in views.

The distribution in space of CS in Yunnan Province from 1980 to 2040 is shown in Figure 7. The spatial pattern of CS in Yunnan from 1980 to 2040 remains generally consistent, with low CS in built‐up land and water and high CS in forestland. Overall, CS is greater in the west and lesser in the east. From 1980 to 2020, the CS primarily increased near existing forestland, with notable increases in Pu'er and Mengla in Xishuangbanna. Low CS areas are mainly concentrated around urban clusters, with their extent continuously expanding, most notably in Kunming. In the latter 20 years, the spatial variation in CS was relatively pronounced. The CS reduction is more pronounced within the main city regions of the central Yunnan urban agglomeration under the two future scenarios. Furthermore, the regional distribution of CS in 2030 and 2040 in the SSP2‐4.5 scenario is comparable to that in 2020. In the SSP1‐2.6 scenario, Yunnan Province's high CS areas expand around already‐existing high CS regions, with notable increases observed in western Yunnan.

### 3.3 Carbon Storage Response to LULC Changes in Yunnan Province

#### 3.3.1 CS Changes Caused by LULC Dynamics

**FIGURE 8:**
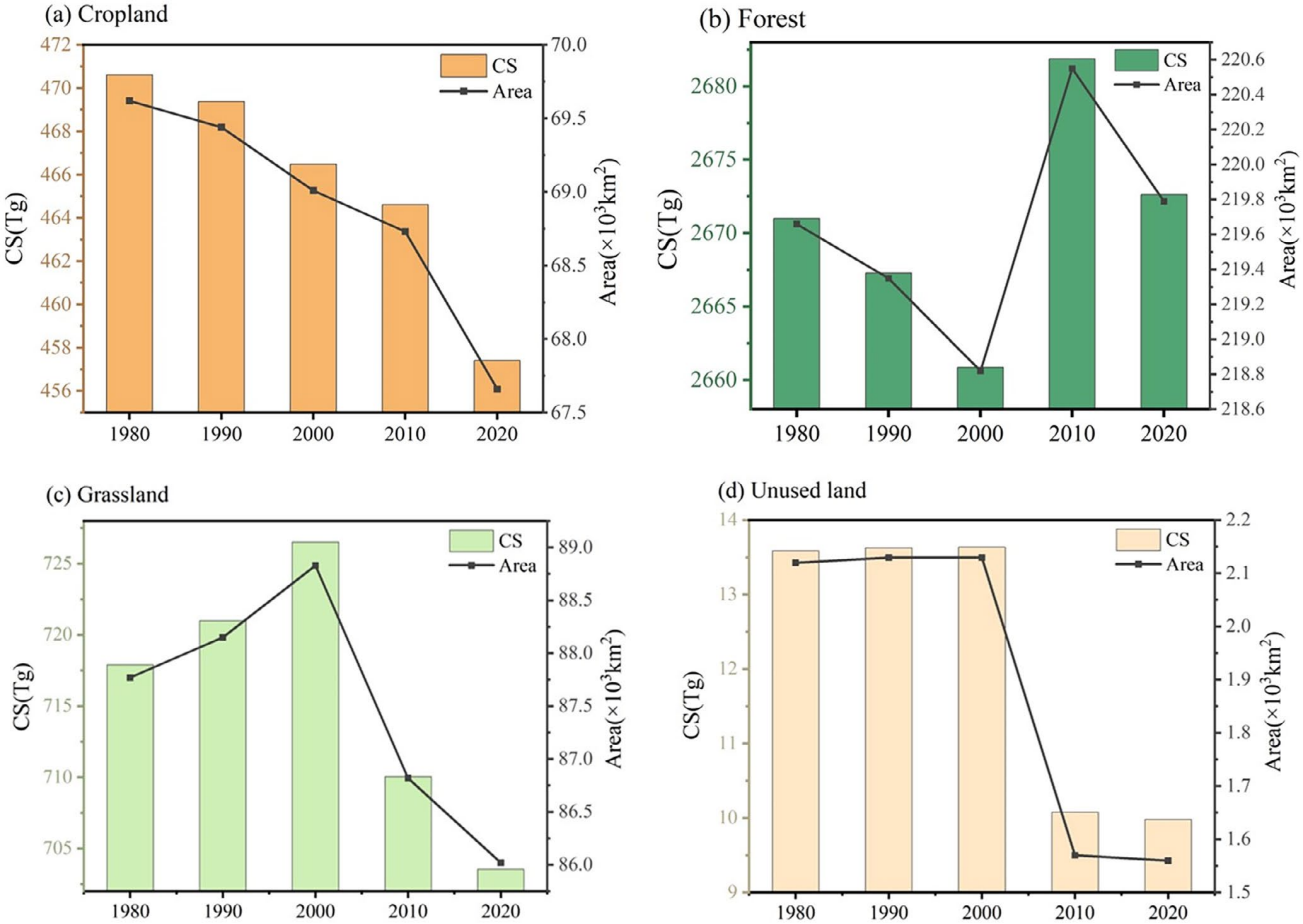
Changes in area and CS for various LULC types. (a–d) represent changes in area and CS for cropland, forest, grassland, and unused land, respectively.

The total CS of a region is correlated with each land classification's area and carbon density because varying LULC categories possess distinct carbon density levels. The changes in each LULC type's area can cause changes in CS, but the effects of area shift on CS vary among different land types (Figure 8) (Table 4). Forestland in Yunnan Province covers the largest area and has the highest carbon density. It is the primary contributor to the total CS in Yunnan, and its area changes exert the most influence on CS, followed by grassland and cropland. From 1980 to 2020, the CS of forestland initially decreased, then increased, and finally decreased again due to changes in its area. The significant increase in area from 2000 to 2010 led to an increase in CS by approximately 21 Tg, while the reductions during other periods were smaller. Overall, from 1980 to 2020, forestland CS increased by 1.61 Tg. Grassland CS initially rose and then declined, resulting in a reduction of 14.35 Tg in 2020 compared to 1980. The continuous decrease in cropland area led to a CS reduction of 13.20 Tg. Unused land, with its relatively small area and CS, experienced an overall decrease of 3.61 Tg. Increasing the extent of built‐up land and water will lower the total CS in the study region because these two land types have zero carbon density. The CS of cropland and unused land decreases and subsequently increases in the SSP1‐2.6 scenario between 2020 and 2040 (Table 4). Altogether, cropland CS decreases by 12.98 Tg, while unused land CS increases by 1.25 Tg. Forestland CS increases continuously with the area, resulting in a total increase of 304.29 Tg. Grassland CS decreases continuously with the area, resulting in a total decrease of 201.47 Tg. From 2020 to 2040, cropland CS increases by 89.63 Tg with the increase in area in the SSP2‐4.5 scenario. In contrast, forestland, grassland, and unused land CS continuously decline along with their areas, resulting in total decreases of 49.90 Tg, 82.33 Tg, and 0.10 Tg, respectively. In the historical process of LULC change, the decrease in CS in Yunnan was primarily due to the shrinkage of cropland and grassland. Additionally, the expansion of built‐up land and water also contributed to the decrease in CS. Under the two future scenarios, the alterations in forestland area in Yunnan will have the greatest impact on the variation of CS.

**TABLE 4:** Changes in CS (Tg) of different LULC types in Yunnan during 1980–2040.

Land use type	1980–2020	SSP1‐2.6		SSP2‐4.5	
		2020–2030	2020–2040	2020–2030	2020–2040
Cropland	−13.20	−22.41	−12.98	57.93	89.63
Forestland	1.61	72.18	304.29	−34.49	−49.90
Grassland	−14.35	−27.92	−201.47	−51.46	−82.33
Water	0.00	—	—	—	—
Built‐up land	0.00	0.00	0.00	0.00	0.00
Unused land	−3.61	−0.06	1.25	−0.01	−0.10

**FIGURE 9:**
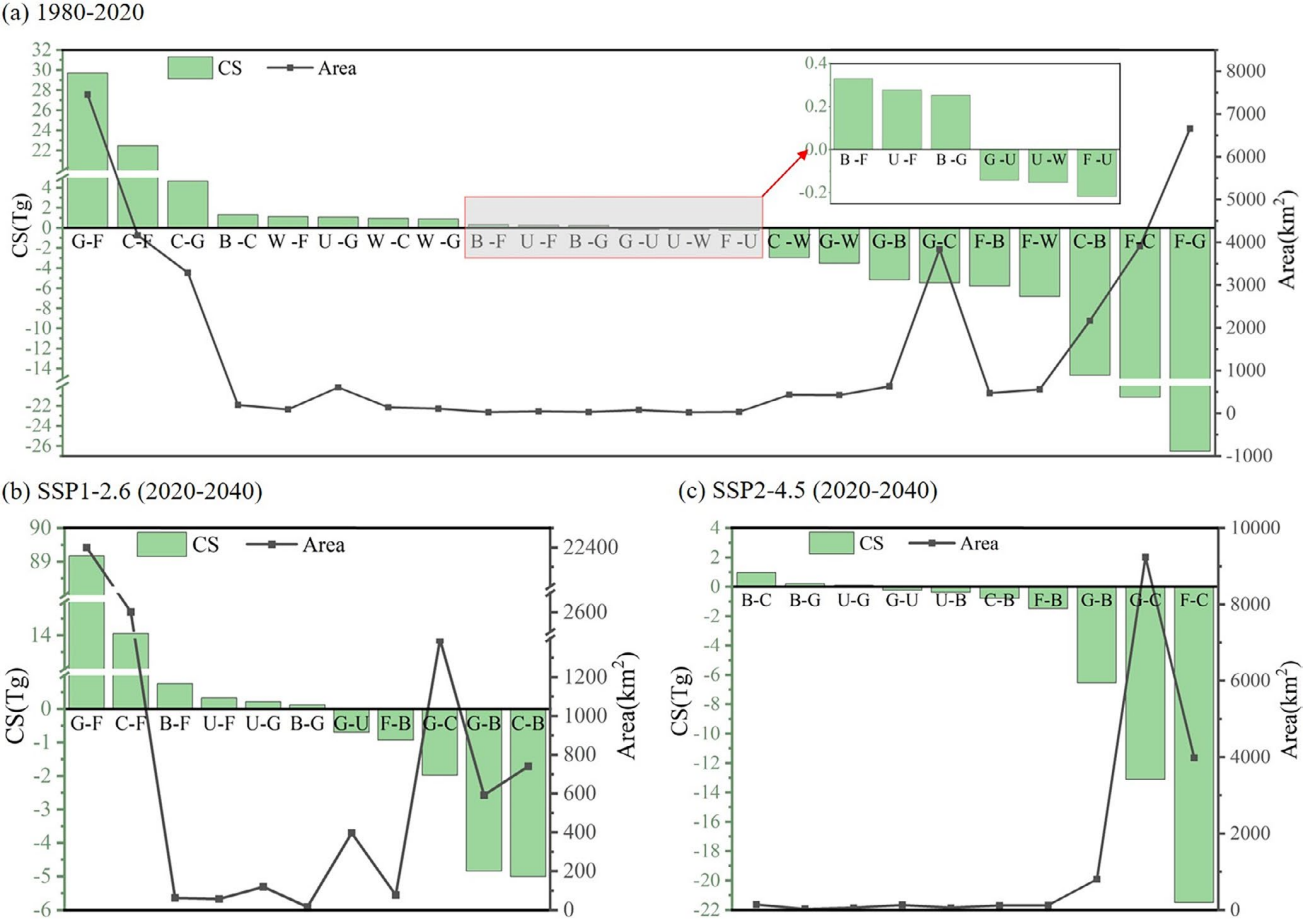
Changes in CS due to transitions between LULC types. (a–c) represent the CS changes caused by LULC type conversions: (a) between 1980 and 2020, (b) between 2020 and 2040 under the SSP1‐2.6 scenario, and (c) between 2020 and 2040 under the SSP2‐4.5 scenario. C, cropland; F, forestland; G, grassland; W, water; B, built‐up land; U, unused land; B‐G indicates the LULC type change from built‐up land to grassland, while G‐B indicates the change from grassland to built‐up land.3.3.2 CS changes zones in Yunnan Province.

The conversion between land types within a region inevitably leads to changes in CS (Figure 9). From 1980 to 2020 in Yunnan Province, the conversion of grassland to forestland (7459.32 km^2^), cropland to forestland (4163.95 km^2^), and cropland to grassland (3289.62 km^2^) increased CS by 29.70 Tg, 22.49 Tg, and 4.67 Tg, respectively. Conversely, the conversion of forestland to grassland (6660.50 km^2^), forestland to cropland (3914.01 km^2^), and cropland to built‐up land (2168.42 km^2^) decreased CS by 26.52 Tg, 21.14 Tg, and 14.66 Tg, respectively. The conversion areas between forestland, cropland, and grassland are substantial, and these land classes exhibit relatively high carbon densities. However, the reciprocal transfers among them result in comparable increases and decreases in CS, thus the mutual exchanges do not lead to significant changes in total CS of Yunnan. From 2000 to 2020, the built‐up land area in Yunnan rose markedly, mostly from the conversion of cropland, with smaller portions from forestland and grassland. These land types have much higher carbon densities than built‐up land, so the expansion of built‐up land caused a decline in total CS. Particularly, the substantial cropland transformed to built‐up land affected a more pronounced reduction in CS. From 2010 to 2020, in parts of river basins such as the Lancang River, the transformation of high‐carbon‐density forestland, grassland, and cropland to low‐carbon‐density water also contributed to a notable loss in CS. Under the SSP1‐2.6 scenario from 2020 to 2040, the main land type conversions are grassland to forestland (22,399.98 km^2^) and cropland to forestland (2602.14 km^2^), which increase CS by 89.17 Tg and 14.05 Tg, respectively. Conversely, the conversions of cropland to built‐up land (740.38 km^2^), grassland to built‐up land (591.90 km^2^), and grassland to cropland (1393.74 km^2^) decrease CS by 5.00 Tg, 4.84 Tg, and 1.98 Tg, respectively. From 2020 to 2040, the main land type conversions under the SSP2‐4.5 scenario are forestland to cropland (3980.96 km^2^), grassland to cropland (9240.10 km^2^), and grassland to built‐up land (800.97 km^2^), which decrease CS by 21.50 Tg, 13.11 Tg, and 6.55 Tg, respectively.

**FIGURE 10:**
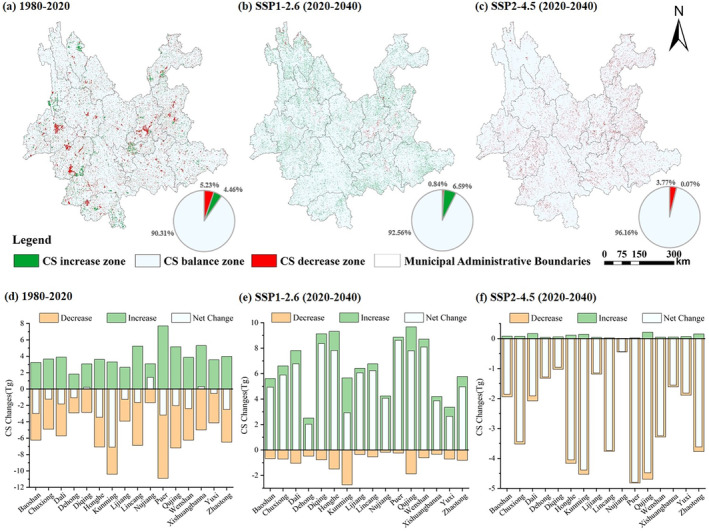
CS changes in Yunnan Province. (a–c), Spatial distribution of CS changes in Yunnan Province. (d–f) CS changes in various cities of Yunnan Province.

In this study, we delineated regions of CS increase zone, CS balance zone, and CS decrease zone based on changes in CS (Figure 10). It can be observed that during 1980–2020, CS balance zones accounted for 90% of the total regional area. CS decrease zones (5.23%) were greater than CS increase zones (4.66%). Overall, there was little change in CS across the province, with changes occurring in scattered and diverse regions. In Baoshan and Lincang, there are relatively large contiguous CS decrease zones. This is primarily a result of grassland being converted to cropland, which leads to a reduction in CS. In urban and township areas such as Kunming, CS decrease zones are primarily attributed to the transition from cropland, grassland, and forestland to built‐up land, resulting in a decrease in CS. In areas such as Nujiang and Xishuangbanna, there are relatively large contiguous CS increase zones, mostly attributed to transformation of grassland into forestland, resulting in an increase in CS. In the northeastern part of Diqing, CS increase zones result from unused land being converted to grassland, leading to an increase in CS. From 1980 to 2020, in Yunnan Province, only the Diqing, Nujiang, and Xishuangbanna regions experienced a net increase in CS, while Kunming had the largest net decrease in CS.

Looking at the projected changes in CS in Yunnan Province by 2040 under two scenarios, in the SSP1‐2.6 scenario, although changes are scattered across the region, CS increase zones (6.59%) are significantly larger than CS decrease zones (0.84%). CS increase zones are scattered throughout the province, with large areas of cropland and grassland being converted into forestland to form CS increase zones. CS decrease zones are mainly in urban and township areas, primarily owing to the alteration of ecological land to built‐up land. The total change in CS for all prefecture‐level cities shows growth under the SSP1‐2.6 scenario. Puer and Diqing have the highest increases in CS, while Qujing and Honghe show significant changes in both increases and decreases, making them noteworthy areas of concern. Under the SSP2‐4.5 scenario, CS decrease zones (3.77%) are significantly larger than CS increase zones (0.07%). In this scenario, not only is high‐carbon‐density forestland and grassland extensively converted to relatively low‐carbon‐density cropland, but low‐carbon‐density built‐up land also occupies ecological land, forming large carbon source areas. The total change in CS for all prefecture‐level cities shows a decrease in the SSP2‐4.5 scenario, with Puer, Qujing, Kunming, and Honghe experiencing the most significant reductions.

## 4 Discussion

### 4.1 Historical LULC Changes Reduced CS

LULC and CS changes from 1980 to 2020 in Yunnan Province were relatively stable, with greater changes observed in the latter 20 years compared to the first 20 years. Forestland in Yunnan Province is the primary contributor to CS, while the main reason for CS reduction is the expansion of built‐up land area.

**FIGURE 11:**
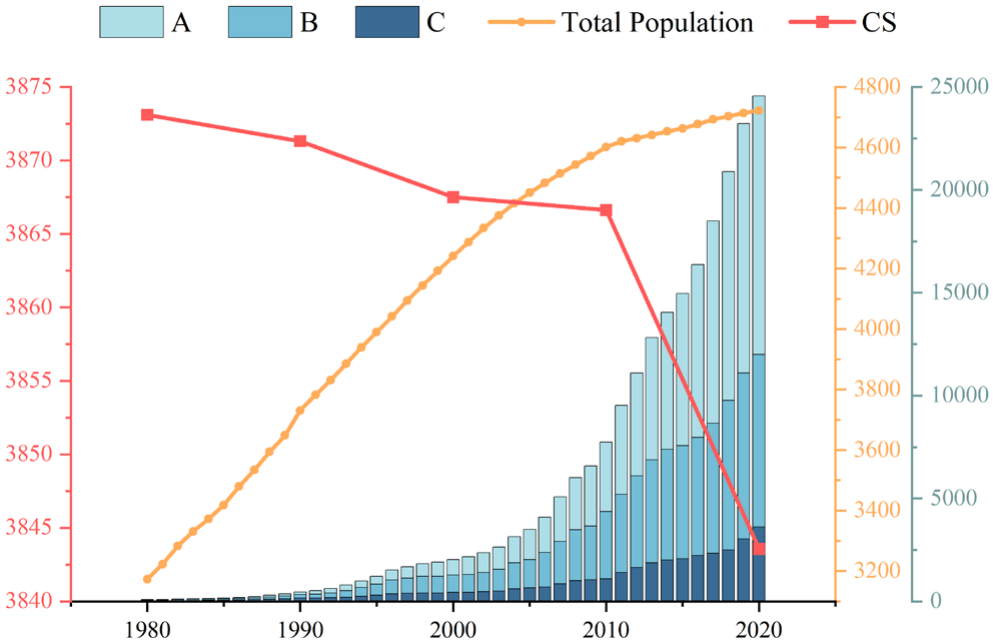
Changes in Yunnan Province from 1980 to 2020 in terms of GDP of primary, secondary, and tertiary industries (in billion RMB), total population (in tens of thousands), and CS (Tg). (A) GDP of primary industries; (B) GDP of secondary industries; (C) GDP of tertiary industries.

Yunnan has large forestland areas with the highest carbon density (Yu et al. 2022), and forestland CS accounts for 68% of the total CS. Its area changes exert the most significant influence on the total CS, followed by grassland and cropland. Since 2000, Yunnan has implemented projects like “Grain for Green” and “Natural Forest Protection,” effectively protecting forestland areas. From 2000 to 2010, the cropland areas and grassland areas reduced, while the low‐carbon‐density built‐up land expanded. However, the overall decrease in Yunnan Province's total CS was minimal due to the relatively larger increase in forestland, which possesses the highest carbon density. From 1980 to 2020, Yunnan Province's CS steadily declined, with a total reduction of 29.55 Tg. Persistent encroachment of built‐up land on high‐carbon‐density areas is a significant reason for the decrease in CS. Between 1980 and 2020, built‐up land expanded 2.75 times, closely correlated with demographic expansion and economic progress in Yunnan Province. Relevant data from the *YUN NAN STATISTICAL YEARBOOK* indicates that Yunnan Province's population increased continuously from 31.73 to 47.22 million between 1980 and 2020. The GDP expanded from 8.427 billion yuan to 2455.572 billion yuan. The share of the tertiary sector's GDP rose from 17% in 1980 to 51% in 2020 (Figure 11). Especially from 2010 to 2020, Yunnan Province's GDP increased rapidly, accounting for 68.73% of the total increase over 40 years. In this time frame, the built‐up land area sharply increased, representing 54.91% of the overall increase over 40 years. This is because quick economic advancement has driven the extensive development and utilization of land. Due to the growing population and fast‐paced economic advancement in Yunnan Province, the requirement for built‐up land for housing, industry and commerce, transportation, tourism, and other purposes has continuously increased in urban and township areas. The ongoing encroachment of built‐up land on surrounding croplands and other high‐carbon‐density ecological lands has caused a decline in regional CS, which matches prior studies (Jin et al. 2015; Wang, Li, Li, et al. 2022). Especially as the provincial capital of Yunnan, Kunming has a dense population and a booming economy. It has the largest net reduction in CS between 1980 and 2020. In contrast, Nujiang, with relatively lower economic development and less development intensity, had the largest net increase in CS among the regions in Yunnan.

It's worth noting that there was a notable rise in water area in Yunnan Province from 2000 to 2020, mostly around certain sections of rivers like the Lancang River. Increases were notably observed around the Xiaowan Dam (completed in December 2008) and the Nuozhadu Dam (completed in June 2014). Because the water in this study includes natural terrestrial water bodies and water conservancy facility land, the rise in water area may be due to the construction of hydroelectric stations. This construction causes a growth in the water surface area behind dams, as well as an increase in land used for hydraulic facilities. Although hydroelectric stations can provide abundant water resources for the region after construction, promote vegetation growth, and potentially increase CS (He et al. 2024), the construction of these stations can disrupt the local ecosystem and cause a decline in CS. In this research, the CS calculated using the LULC data and the carbon density data for every category for that year only represents the situation of that year. Since water bodies have a carbon density of zero, the increase in water area in this study results in a decrease in CS.

In summary, ecological projects like the “Grain for Green” and “Natural Forest Protection” have to some extent, slowed down the decline of CS in Yunnan Province over the past four decades. However, rapid economic development over the last decade, coupled with significant increases in built‐up land and major construction projects such as hydroelectric dams, has still led to a decrease in Yunnan Province's CS.

### 4.2 Carbon Storage Response to LULC Changes Under SSP‐RCP Scenarios

From 2020 to 2040, significant changes in LULC and CS in Yunnan Province will occur within various future scenarios. Under the SSP1‐2.6 scenario, which prioritizes sustainable development and environmental protection with strict government ecological conservation policies, the climate is relatively stable, conducive to ecosystem protection and restoration. In this scenario, high‐carbon‐density forestland in Yunnan Province increases significantly, leading to a substantial rise in CS. The future increase in carbon storage can effectively reduce greenhouse gas emissions and mitigate climate change, potentially creating a positive feedback loop that promotes further ecosystem restoration. In contrast, under the SSP2‐4.5 scenario, lower policy constraints, higher greenhouse gas emissions, and significant climate change adversely affect the health and functionality of ecosystems. In this scenario, the reduction of high‐carbon‐density forestland and grassland, along with the expansion of low‐carbon‐density built‐up land, leads to a decline in CS. This, in turn, results in increased greenhouse gas concentrations and rising temperatures, which may create a negative feedback loop that exacerbates climate change. Comparing the two scenarios, the SSP1‐2.6 scenario can meet the future land use needs of the region while increasing CS, and it also helps to mitigate climate change. This indicates that implementing stringent ecological protection policies in Yunnan Province can effectively increase the forestland and grassland areas, enhance regional CS, and mitigate regional climate change. Promoting sustainable development in Yunnan Province will better serve China's carbon neutrality goals. This in line with the findings on the various carbon pools in terrestrial ecosystems of China under different SSP‐RCP scenarios (Ding and Sun 2023).

In summary, high‐carbon‐density forestland dominates the changes in CS in Yunnan Province. Converting low‐carbon‐density land types to high‐carbon‐density land categories can raise CS, and conversions between land types with significant differences in carbon density can result in substantial changes in CS. Similar findings have also been made by earlier research (He et al. 2023; Tian et al. 2022; Wei, Zhang, et al. 2023; Wu and Wang 2023). In the future, if Yunnan implements high‐intensity‐related ecological policy management, the area of forestland and grassland will rise significantly, thereby increasing the regional CS. Therefore, through reasonable land management and strict ecological protection, consolidating and strengthening ecological projects such as “Grain for Green” can help increase the area and improve the quality of forestland in Yunnan Province, which is beneficial to increasing the CS.

### 4.3 Suggestions and Insights

Accurately investigating the ecological changes within the area provides key information for policymakers in developing regional sustainable development plans (Dong et al. 2018). From 1980 to 2020, the Nujiang, Diqing, and Xishuangbanna regions in Yunnan Province were net CS increase zones, with abundant forest resources, serving as important ecological barrier areas in Yunnan. It is essential to continue strengthening ecological protection in these areas. In contrast, the economic core of Kunming in Yunnan Province has a dense population and swift economic growth, leading to the largest net decrease in CS. In the future, balancing economic progress and ecological conservation in Kunming will be a central concern. By 2040, under two future scenarios, there will be significant differences in CS in Yunnan Province due to different LULC changes. In the sustainable development scenario SSP1‐2.6, under the control of high‐intensity policies, the low‐carbon‐density grassland and cropland in Yunnan Province will significantly transform into high‐carbon‐density forest, leading to an increase in carbon storage and potentially creating a large carbon sink. In contrast, under the low‐intensity policy scenario SSP2‐4.5, forest and grassland in Yunnan Province are likely to convert into cropland with lower carbon density, while built‐up land will significantly encroach on ecological land, resulting in a decrease in carbon storage and potentially turning Yunnan Province into a significant carbon source. Due to the larger areas and better economic development in regions such as Pu'er, Qujing, and Honghe, where the degree of LULC change is higher, the changes in CS in these areas are particularly pronounced and warrant special attention within Yunnan Province. While developing the “one circle, one core, two clusters, and one belt” model, efforts should also be made to strengthen the protection and construction of wetlands around plateau lakes such as Dianchi and Fuxian Lake to improve the urban ecological environment. In future development, Yunnan Province should continue to advance forest protection and national land greening initiatives, strengthen forest resource management (Tong et al. 2020), and enhance the construction and protection of plateau lake wetlands in northwestern Yunnan. By optimizing the spatial development layout of towns, Yunnan can achieve development through conservation. Leveraging its geographical advantages to promote regional sustainable development is also significant for China in achieving its carbon neutrality goals.

### 4.4 Limitations and Prospects

We integrate InVEST‐PLUS‐LUH2 to reveal the historical and future LULC changes in Yunnan Province under various climate and socio‐economic development scenarios and their impacts on the spatio‐temporal patterns of CS. Nevertheless, certain restrictions and unknowns still exist. Firstly, the InVEST model uses static carbon densities of various land categories to estimate the regional CS. It neglects the differences in carbon density among different forest stands and plant age structures (Fan et al. 2023). Additionally, Yunnan Province is a large region with a complex and diverse climate, and there exist variations in carbon density within identical land categories across different areas. The carbon density data we utilized includes the global biomass carbon density map, which was compiled by integrating multiple published and rigorously validated data sources, as well as the global soil dataset, derived from interpolating ground measurement data. The accuracy of these data has been verified. Therefore, we used these data to estimate carbon stocks (CS), minimizing the uncertainty in our estimates to the greatest extent possible. To enhance the precision of the assessment, it is advised that future researches gather more precise and comprehensive data on carbon density using field measurements and other techniques. Secondly, we used the PLUS model combined with LUH2 data and various socioeconomic and natural variables to imitate future LULC data. Although the model has high precision of simulations, the model simulates the future according to current land transformation rules. However, future LULC alterations are shaped by numerous elements, including policies, and future transformation rules may alter (Guo et al. 2023). Therefore, future studies should comprehensively consider policy elements, like regional development plans, on future LULC. The accuracy of simulating future LULC alterations can be further improved by innovating methods to adjust LULC conversion rules and model parameters. Thirdly, the LUH2 dataset does not include water change data. In our future simulations, we assumed that the water remains unchanged. Although the proportion of water area in Yunnan is not large, it still showed significant changes from 2000 to 2020, which could affect the model's prediction accuracy. Therefore, future studies need to address this issue through methodological improvements and the collection of more data.

## 5 Conclusion

Under the PLUS‐InVEST‐LUH2 coupled prediction framework, this study examines the LULC alterations and their effect on CS in Yunnan Province in historical (1980–2020) and future (2020–2040) scenarios of different climate and socio‐economic development (SSP‐RCP). The research suggests: (1) From 1980 to 2020, the main LULC categories were forestland, grassland, and cropland in Yunnan Province. The built‐up land, although a minor fraction of the entire area, exhibited the largest alteration, rising by 2.75 times. (2) The CS is high in forestland and low in built‐up land, with a spatial pattern that is higher in the west and lower in the east. Over the preceding four decades, Yunnan's CS has continuously reduced by 29.55 Tg. This decline is mostly attributed to population growth and economic progression, which have brought about a growth in low‐carbon‐density built‐up land. This expansion has mostly encroached on cropland, causing a reduction in CS. (3) Under the high‐intensity policy constraints of the sustainable progression scenario SSP1‐2.6, the increase in high‐carbon‐density forestland area will lead to an increase in CS. By 2040, Yunnan Province's CS can increase to 3934.65 Tg, demonstrating significant carbon sink potential. In contrast, under the moderate development pathway SSP2‐4.5 with low‐intensity policy impacts, the reduction in forestland and grassland will lead to a significant decrease in CS. By 2040, Yunnan Province's CS will reduce to 3800.86 Tg. (4) Forestland and built‐up land are the pivotal land categories influencing CS in Yunnan Province. Forestland is the primary contributor to CS, while the continuous enlargement of built‐up land will cause a continuous decrease in CS. Scenario simulations indicate that forthcoming alterations in climate and land utilization will exert considerable influence on CS in Yunnan Province. In these two future scenarios, Yunnan Province may become a carbon sink or a carbon source. Hence, fostering the synergistic development of ecology and economy in Yunnan Province to ensure it becomes a carbon sink in the future is crucial. Therefore, in future development, Yunnan Province should implement stricter ecological protection policies, ensure the effective implementation of relevant policies across all regions, continuously promote national land greening actions, and optimize urban spatial development patterns. This approach will balance development with protection, fully leverage the geographical advantages of Yunnan, enhance the CS and carbon sink functions, and contribute to China's carbon neutrality goals.

## Author Contributions

**Jing Liu:** conceptualization (equal), data curation (equal), formal analysis (equal), methodology (equal), validation (equal), visualization (equal), writing – original draft (equal), writing – review and editing (equal). **Kun Yang:** conceptualization (equal), funding acquisition (equal), project administration (equal), supervision (equal), writing – review and editing (equal). **Shaohua Zhang:** funding acquisition (equal), project administration (equal), resources (equal), supervision (equal), writing – review and editing (equal). **Wenxia Zeng:** data curation (equal), visualization (equal). **Xiaofang Yang:** formal analysis (equal), writing – review and editing (equal). **Yan Rao:** data curation (equal), software (equal). **Yan Ma:** supervision (equal), validation (equal). **Changyou Bi:** supervision (equal), visualization (equal).

## Conflicts of Interest

The authors declare no conflicts of interest.

## Supporting information


Appendix S1.

## Data Availability

The study's data can be accessed at https://doi.org/10.5281/zenodo.13370994. DOI: https://doi.org/10.5281/zenodo.13370994.
