# Supplementary material for: Carbon Storage Response to Land Use/Land Cover Changes and SSP‐RCP Scenarios Simulation: A Case Study in Yunnan Province, China
Source: Ecol Evol. 2025 Jan 8;15(1):e70780. doi: 10.1002/ece3.70780 (PMC11710938; doi:10.1002/ece3.70780)
Supplement: Supplementary file 1 — Appendix S1. [file ECE3-15-e70780-s001.docx]

# Appendix A. Supplementary data

The following is the Supplementary data to this article:

Table A: Reclassification of LUH2 data for LULC simulation.

| **CNLUCC classes** | **LUH2 land classes** |
| --- | --- |
| Cropland | C3 annual crop |
|  | C3 perennial crop |
|  | C4 annual crop |
|  | C4 perennial crop |
|  | C3 nitrogen-fixing crop |
|  |  |
| Forestland | Forested primary land |
|  | Potentially forested secondary land |
|  |  |
| Grassland | Managed pasture |
|  | Rangeland |
|  |  |
| Built-up land | Urban land |
|  |  |
| Unused land | Potentially non-forested secondary land |
|  | Non-forested primary land |
|  |  |
| Water | None |
